# PHTNet: Characterization and Deep Mining of Involuntary Pathological Hand Tremor using Recurrent Neural Network Models

**DOI:** 10.1038/s41598-020-58912-9

**Published:** 2020-02-10

**Authors:** Soroosh Shahtalebi, Seyed Farokh Atashzar, Olivia Samotus, Rajni V. Patel, Mandar S. Jog, Arash Mohammadi

**Affiliations:** 1 Concordia Institute for Information Systems Engineering, Concordia University, Montreal, H3G 1M8 QC Canada; 2 Departments of Electrical and Computer Engineering, and Mechanical and Aerospace Engineering, New York University, New York, 10003 NY USA; 3 London Movement Disorders Centre, London Health Sciences Centre, London, ON Canada; 4 Department of Electrical and Computer Engineering, University of Western Ontario, London, N6A 5B9 ON Canada; 5 NYU WIRELESS center, New York University (NYU), New York, USA

**Keywords:** Computational models, Machine learning

## Abstract

The global aging phenomenon has increased the number of individuals with age-related neurological movement disorders including Parkinson’s Disease (PD) and Essential Tremor (ET). Pathological Hand Tremor (PHT), which is considered among the most common motor symptoms of such disorders, can severely affect patients’ independence and quality of life. To develop advanced rehabilitation and assistive technologies, accurate estimation/prediction of nonstationary PHT is critical, however, the required level of accuracy has not yet been achieved. The lack of sizable datasets and generalizable modeling techniques that can fully represent the spectrotemporal characteristics of PHT have been a critical bottleneck in attaining this goal. This paper addresses this unmet need through establishing a deep recurrent model to predict and eliminate the PHT component of hand motion. More specifically, we propose a machine learning-based, assumption-free, and real-time PHT elimination framework, the PHTNet, by incorporating deep bidirectional recurrent neural networks. The PHTNet is developed over a hand motion dataset of 81 ET and PD patients collected systematically in a movement disorders clinic over 3 years. The PHTNet is the first intelligent systems model developed on this scale for PHT elimination that maximizes the resolution of estimation and allows for prediction of future and upcoming sub-movements.

## Introduction

Age-related neurological movement disorders such as Parkinson’s Disease (PD) and Essential tremor (ET)^1–4^ are expected to become more prevalent as the population of seniors over the age of sixty is expected to increase from 962 million in 2017 to 2.1 billion by 2050, and to 3.1 billion in 2100^5^. Pathological Hand Tremor (PHT) is a common upper-limb motor symptom of several age-related neurological movement disorders and is described as involuntary and pseudo-rhythmic movements^6^ affecting coordination, targeting, and speed of intended motions^7^.

Unlike physiological tremor, which is identified with low amplitude vibrations occurring within the spectral range of 6 to 14 Hz^8^ and affects the performance of individuals in high precision tasks such as robotic surgery^9^, PHT represents higher amplitude motion occurring in the broader frequency range of 3–14 Hz^10^. The repetitive and oscillating nature of PHT differentiates itself from other involuntary movement disorders such as chorea, athetosis, ballism, tics, and myoclonus^11^. Upper-limb tremor significantly limits individuals in performing Activities of Daily Livings (ADLs)^12^. Thus, during the last decade, several techniques and technologies have been proposed in both rehabilitation and assistive domains^13,14^ to compensate for the involuntary movement while promoting the voluntary component of motion. The accuracy of a tremor compensation technology (such as sophisticated wearable exosuits) relies significantly on the efficacy and spectrotemporal resolution of the algorithm, as inaccurate or slow extraction techniques do not allow for proper compensation. PHT consists of the following types of tremor: Rest Tremor (RT) occurring when a limb is relaxed and supported against gravity (commonly observed in PD^15^); Action Tremor (AT) occurring during voluntary contraction of muscles and classified into the subcategories of postural, kinetic, and isometric^16^. Postural and kinetic tremors are commonly observed in ET patients^15^. While postural tremor occurs when an individual voluntarily maintains a position against gravity, such as an outstretched arm, kinetic tremor occurs when a voluntary movement is performed. On the other hand, isometric action tremor exists during muscle contraction against a rigid stationary object such as grasping a solid object that blocks the motion of the limb and changes the length of muscles. It is worth mentioning that although PD tremor is typically characterized by unilateral rest tremor in the spectral range of 4–6 Hz and ET patients commonly show symmetric postural and kinetic tremor in the range of 4–8 Hz, there are many atypical cases (for example PD patients having action tremor and ET patients having rest tremor) that PD and ET share overlapping features^17^.

PHT therapies such as oral medication, therapeutic lesions, Gamma-knife radiosurgery, and Deep Brain Stimulation (DBS)^18^ focus on alleviating tremor severity and improving arm functionality^19–21^. Although efficacy is reported for these PHT interventions, about 25% of patients are unresponsive or experience short-term suboptimal response^12,22,23^, and adverse side effects are also commonly observed. The severity and characteristics of PHT are assessed and monitored^3,24–34^ through recording and processing of hand motions in clinical settings while performing different tasks. This information has also been used to tailor dosing and regimen of therapy, such as botulinum toxin type A (BoNT-A) injections^35^. However, a major remaining challenge in assessing action tremor is the processing and separation of voluntary and involuntary components, which is not accurate using conventional approaches. Thus, some of the recently-developed techniques and therapies (e.g. BoNT-A therapy)^35–39^ that finely tailor dosing and muscle selection for targeted therapy based on accurate signal monitoring, would not be feasible for patients with prominent action tremor.

Recently, robotic rehabilitation and assistive technologies^40–44^ have attracted a great deal of interest due to their promising performance in compensating the involuntary tremor in both rehabilitation and assistive settings. Such technologies^40,42,45,46^ are mainly developed to remove (damp out or compensate) the tremor and assist patients in performing their voluntary movements^41,47–49^. However, the performance and efficacy of such technologies is directly linked to the accuracy of tremor estimation, which is a nonstationary, nonlinear, and uncertain signal processing challenge. Thus, accurate PHT elimination (and possibly prediction) is important for both robotic rehabilitation and assistive technologies, and also for different clinical applications. This will allow proper delivery of the expected assistive actions at the right time of oscillation, and also guarantees the required level of safety. In particular, for these settings, it is essential to have zero or minimal phase lag in the estimation and extraction of tremor to effectively and simultaneously generate a counteracting force field. It is shown^50^ that a phase-lag as low as 20 *ms* in the signal filtering part noticeably degrades the system performance. As the frequency content of pathological tremor can be close to that of the voluntary component, having zero or minimal phase-lag is a major challenge. However, this would be feasible if a robust and generalizable model of tremor is designed that can encapsulate nonlinear temporal dependencies between sub-movements during task performance.

Despite the crucial need for PHT elimination techniques and the numerous research advancements on this topic^51–55^, including our previous work^13,14^, there is an unmet need for a reliable, adaptable, and generalizable processing framework that can be directly translated to clinical settings for estimation, extraction, and prediction of action tremor with high spectrotemporal resolution. Among previously published studies, Band-limited Multiple Fourier Linear Combiner (BMFLC)^51^, Extended-BMFLC (EBMFLC)^13^, and Wavelet Adaptive Kalman Estimation (WAKE)^14^ frameworks demonstrate the most successful current state of the available approaches. The FLC-based methods, e.g., BMFLC and EBMFLC, are aimed at deriving linear mixing models for the spectral contents of the motion signal to distinguish and separate the two components. The BMFLC employs fixed predefined values to identify the spectral range of PHT and is aimed at attenuating those contents to derive the voluntary component. The EBMFLC, on the other hand, tries to adaptively identify and remove the spectral range of the PHT, which has shown superior performance compared to its counterpart. Recently, the WAKE framework showed significant improvement in performance in comparison to all existing techniques for PHT elimination. WAKE exploits Wavelet transforms and Kalman filtering to obtain a spectrotemporal representation of motion signals to separate the voluntary and involuntary components. Existing methods including EBMFLC and WAKE, share a similar characteristic by assuming that the spectral contents of voluntary and involuntary components are distinct and one could be derived by removing the other from the measurement signal. However, this assumption is not always realistic and has resulted in limited performance of the designed techniques and also hindered their clinical translation. In fact, the frequency contents of the voluntary and tremor components are not completely distinct from each other, and the overlapping of spectral contents is quite natural. Thus, considering all high-frequency components as tremulous motion results in inferior performance of the technique, which may fail to follow voluntary changes in the direction and frequency context of motion. Such behaviour can dramatically degrade the performance of any assistive technology that is supposed to react to the changes in motor intent, in an agile manner. This can significantly affect the estimation of involuntary components (through misinterpretation as involuntary motion the high frequency content of voluntary motion caused by nonlinear and nonstationary changes). This can directly affect the regimen of advanced new therapies that are tailored based on such measures. We believe that the above-mentioned issue is an important contributing factor for the limited performance of previous methods.

Besides concerns regarding the computational power and capacity of existing frameworks for an ultimate predictive model, there is a need for characterizing tremor based on a sizable inclusive dataset, that covers possible pathological variations causing diverse types of tremor signals in terms of spectrotemporal behavior, dynamic nature, temporal dependencies, and sub-movements. Without such a data atlas, conservative and impractical assumptions would be considered to define a ground truth reference for designing and validating the techniques. We argue that due to the high degree of inter- and intra-subject variability of tremor characteristics, the solutions designed and validated based on a limited dataset may not be generalizable for translation to the clinic and ADL for PD and ET patients. Thus, building a representative and sizable dataset coupled with designing a predictive model with high spectrotemporal capacity are critical for developing a PHT removal and prediction framework that is robust to inter- and intra-subject variability of tremor characteristics. This unmet need is addressed and discussed in this paper by utilizing a novel data-driven deep neural network modeling technique with a unique tremor extraction capacity augmented by predictive power and trained based on our unique dataset. The dataset includes kinematic motion recordings of 81 PD and ET patients (about hours of recordings) collected in a movement disorders research laboratory based on a rigorous systematic protocol. This is the largest dataset known to date permitting the generation of a strong high-capacity model. Developing a PHT extraction framework by employing large datasets, which include recordings of an extensive number of patients over long periods of time, takes a significant range of possible variations in the characteristics of tremor into account, adds more generalization to the framework, and makes the technique adaptable to inter and intra-patient variabilities, nonstationary and nonlinear behaviours of tremor signals. In other words, utilization of such larger dataset allows the network to more securely avoid the curse of overfitting to the training samples by observing a more diverse range of possibilities in the PHT behavior. The proposed model, referred to as PHTNet, is a real-time and assumption-free neural model developed based on bidirectional recurrent neural networks. PHTNet can process measurement signals in both online and offline fashion and provides one-sample-ahead (one sample ahead) predictions on the voluntary component of hand motion signals, which is the ultimate temporal resolution for this application. It should be noted that the time resolution of signal processing plays a very important role when the control loop gain and the frequency of activations in a control system are high (such as the robotic rehabilitation devices)^56^. Therefore, one sample ahead-of-time prediction can play a very important role for compensation of external disturbances and achieving the control goals. On the other hand, a major difference between the proposed intelligent data-driven tremor extraction model and conventional filters is that since it is supported by the deep modeled connectivity in the dataset, it not only can remove phase latency but also has a one sample prediction. It is worth noting that phase lag (even in the order of 10 ms) can easily make a high-gain rehabilitation robot unstable which can sacrifice safety^13,56–59^. Therefore, the proposed PHTNet, which not only removes the lag but also enhances the time resolution, provides a significant phase benefit, which is imperative for the control algorithm of rehabilitation and assistive technologies.

To address the need for a valid ground truth of the voluntary component of action tremor signals when training the PHTNet, through a novel design, we have employed a mixture of synthesized voluntary components and recorded rest and postural tremor signals from PD and ET patients to teach the network to distinguish between the two motor behaviours. It is worth noting that this study is not made based on any potential differences between the characteristics of tremor in PD and ET. The data from two population of patients is collected to get a large variety of tremor characteristics and to empower our algorithm for a large population of users. One of the differences between PHTNet and previous works in the literature is that we do not bound the characteristics of PHT into a few commonly used parameterized assumptions in the spectral domain, as this is a highly dynamic feature. Instead, we employ a data-driven approach, PHTNet, which learns how to internally evaluate the separation criteria and employ them for extraction and prediction of the voluntary component through several training samples, without explicitly introducing any characteristic of or assumptions on the tremor. Moreover, the devised training strategy of PHTNet teaches the network to model the nonlinear short-term and long-term temporal dependencies and minimize the error between the output sequence and the forthcoming samples of the ground truth signal to equip PHTNet with predictive behaviour, which, for the first time, presents an ultimate temporal resolution.

It is worth noting that the PHTNet is not proposed as a tremor treatment procedure, rather, it is developed to enhance the efficacy of current tremor management methodologies and also improve the quality of services delivered to the patients by robotic assistive devices. In other words, PHTNet can be put into practice to extract the voluntary and involuntary components of action tremor signals with a high spatiotemporal resolution to help the neurologists with objectively assessing the characteristics of tremor over the course of action and time, needed to specify the dosage and plan of prescribed medications. In addition, PHTNet can be directly employed by robotic assistive and rehabilitative technologies to enhance the quality of delivered assistive and rehabilitative services by maximizing the performance in reducing the tremor and stabilizing the motion, and by minimizing the risk of amplifying the tremor component by the device. Failure in precisely removing the tremor component in the input signals to the assistive devices can result in abrupt and unpredictable force profiles generated by the device, which can sacrifice safety. Details can be found in our previous papers in^13,60^.

## Methods

This section describes the basics of the proposed PHTNet, as well as the systematic data collection strategy employed to design and train the network.

### Dataset

This dataset was collected from 81 PD and ET individuals who participated in a single-centre. The study protocol was approved by the Western University’s Health Sciences Research Ethics Board (REB#: 104584 and 107433) at the London Movement Disorders Centre in London, Ontario, Canada. The study protocol is registered with the “www.clinicaltrials.gov” registry (Identifiers: NCT02551848 and NCT02668497). All experiments were conducted in accordance with the Declaration of Helsinki, as well as the Tri-Councel Policy Statement of Ethical Conduct for Research Involving Humans in Canada. The ethics committee provided full board approval for this study protocol and the consent procedure was approved as required in the documentation checklist, submitted with the full study protocol. Demographics data of the PD and ET group are tabulated in the supplementary material. All participants were recruited through the Movement Disorder Centre, at the University Hospital, London, Ontario, Canada. All participants provided written informed consent regarding their participation in the study. The participants recruited met the inclusion/exclusion criteria^35,39^. First participant’s first visit and last participant’s last visit occurred in March 2014 and January 2018, respectively.

A convenience sampling of 119 PD and 131 ET upper-limb tremor assessments were utilized to develop PHTNet. The PD group included 47 patients, 8 females, and 39 males, with an average age of $71.51\pm 7.63$, where 26 of them were de novo patients. 14 and 35 patients were recorded bilaterally and unilaterally, respectively. 45 patients were assessed in two sessions with a time interval of 6 weeks and only 2 patients participated once. The ET group included 34 patients, 13 females, and 21 males, with an average age of $69.8\pm 6.12$. This group included 22 de novo patients and the whole ET group was assessed bilaterally; 3 patients participated only once and the rest were assessed twice, with a time interval of 6 weeks.

**Figure 1:**
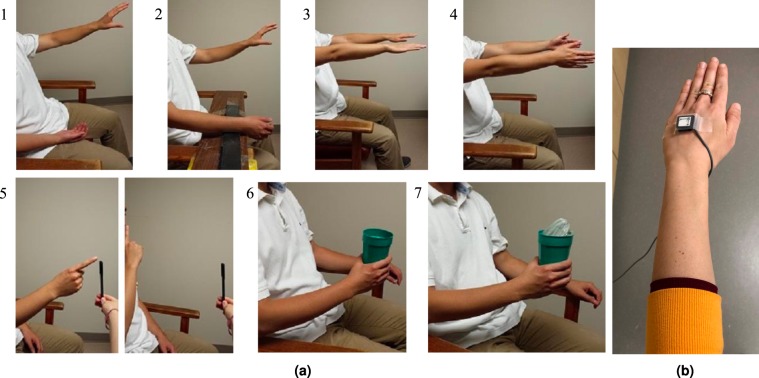
(**a**) Illustration of the 7 scripted tasks performed by PD and ET patients for each tremor assessment. Please note that, these are representative pictures (not including any patients). 1) Rest-1; 2) Rest-2; 3) Posture-1; 4) Posture-2; 5) Action tremor (repetitive finger to nose motion); 6) Load-1 (empty cup); 7) Load-2 (1-lb weight in the cup). **(b)** Placement of the 3-axis accelerometer sensor on the dorsum of a hand.

Kinematic analysis of upper-limb tremor was conducted by having participants perform a series of seven scripted tasks each held for 20 seconds over three trials, as previously described^35,39^ and illustrated in Fig. 1a: two rest positions with the forearm supported on the lap (“Rest-1”) or supported on a board (“Rest-2”), two postural positions with the arms pronated outstretched with palms facing downwards (“Posture-1”) or with arms outstretched and palms facing each other (“Posture-2”), two weight-bearing tasks with participants holding an empty cup (“Load-1”) and holding a cup with a 1-lb weight (“Load-2”), and one kinetic/action task where participants conducted a repetitive finger-to-nose action. Thus, 6 of the 7 tasks captured PHT in a static position (denoted as “static tremor”) and the finger-to-nose dynamic task provided “action tremor” data. An inline 3D accelerometer sensor (#317A Noraxon U.S.A Inc.) was placed on the back of the hand, as illustrated in Fig. 1b, to capture hand tremor in real-time using TeleMyoTM 2400T G2 at 1500 Hz and transmitted to a computer running MyoResearch XP Version 1.08.0951,62. In total, 87.5 hours of data were used in this work collected from 81 patients (3 channels for each patient, 7 minutes per assessment, and 250 tremor assessments in total).

### Data preparation

To prepare the dataset for development of the PHTNet, all the recorded signals were downsampled to 100 Hz. Based on the Nyquist sampling theorem^61^, for full reconstruction of a sampled signal, one needs to set the sampling frequency at least to double that of the maximum meaningful frequency of the signal. It is worth noting that although downsampling may generally impose distortions on the signals, in the case of tremor removal with the frequency of interest generally being less than 15 Hz, downsampling to 100 Hz would impose minimum to no distortion to the spectral range of interest in the hand motion signals, while at the same time it avoids imposing excessive computational costs on the system. Thereafter, the dataset was divided into three sets for training, validation, and testing of the network. Table 1 explains the categorization of the data used for training and evaluation of PHTNet. It is worth mentioning that to impose harsh evaluation conditions on the PHTNet and strictly avoid leakage of information, directly or indirectly, from training set to the validation and test sets, the training, validation, and test sets are formed based on subjects. In other words, the recordings of 49, 16, and 16 subjects are respectively employed to form each of the development sets. Furthermore, due to the availability of a large dataset for development of PHTNet, three sets for training, validation, and testing are formed.

**Table 1:** Categorization of the data for training, validation and testing purposes.

**81 Patients (250 Tremor Assessments)**		
20% Test Data (16 subjects)	20% Validation Data (16 subjects)	60% Training Data (49 subjects)
• Static tremor was used for quantitative evaluation	• Static tremor was used for validation of the model	• Static tremor was used for training the network
• The action tremor of the three sets was employed to qualitatively monitor the performance of the network.		

Based on the aforementioned motivations in employing pseudo-synthesized data, we addressed the need for a valid ground truth in action tremor signals by synthesizing the voluntary component and mixing it with static tremor signals. The voluntary component was a sinusoidal signal with random amplitude, frequency, and phase, which was modeled as

1 $${m}_{v}^{({\rm{GT}})}(t)=a\,\sin \,\mathrm{(2}\pi ft+\phi ),$$

where amplitude, frequency and phase follow uniform distributions, i.e., $a\sim {\bf{U}}(0,0.25)$, $f\sim {\bf{U}}(0,3)$ Hz, and $\phi \sim {\bf{U}}\mathrm{(0},\pi )$, respectively. Since we assume an additive model for the voluntary and involuntary components to build the motion signal, the synthesized voluntary component was mixed with the experimentally-collected static tremor signals from PD and ET participants, which were scaled to the range of [0, 0.5], and PHTNet was fed with a pseudo-synthesized action tremor signal in the range of [0, 1]. Moreover, in this model (Eq. (1)), we assume that the frequency contents of the voluntary motion are spread over the range of [0, 3] Hz, which is a reasonable assumption as we do not expect very fast hand motions from the ET and PD participants, due to the rigidity and stiffness of the muscles. It is worth noting that this assumption is with regard to the voluntary component and does not impose any assumption on the involuntary component. Moreover, the spectral range for the pseudo-synthesized voluntary hand motion, compared to conventional methods, is a more relaxed assumption as typically this range is taken to be up to 1 Hz. Although the spectral range of [0, 3] Hz takes a wide variety of motions into account, it does not imply that no tremulous activity occurs in this range and this could be marked as one of the main advantages of this work over conventional methods.

### Internal architecture of the PHTNet

Machine learning is defined as a study of statistical and mathematical models, which enable a computer to capture the behaviour of a certain phenomenon without explicit instructions. Conventional machine learning methods are based on hand-crafted and user-engineered techniques developed to transform and represent raw data in a format which is perceivable by mostly-linear, or linear-in-parameter mathematical models. Performance of traditional machine learning methods, however, is normally restricted due to their limited modeling/learning capability. In addition, conventional machine learning methods require domain expertise and careful system design in order to have an acceptable performance. Therefore, representation learning methods^62^ are introduced and developed such that the intrinsic patterns of input data are automatically inferred and extracted.

**Figure 2:**
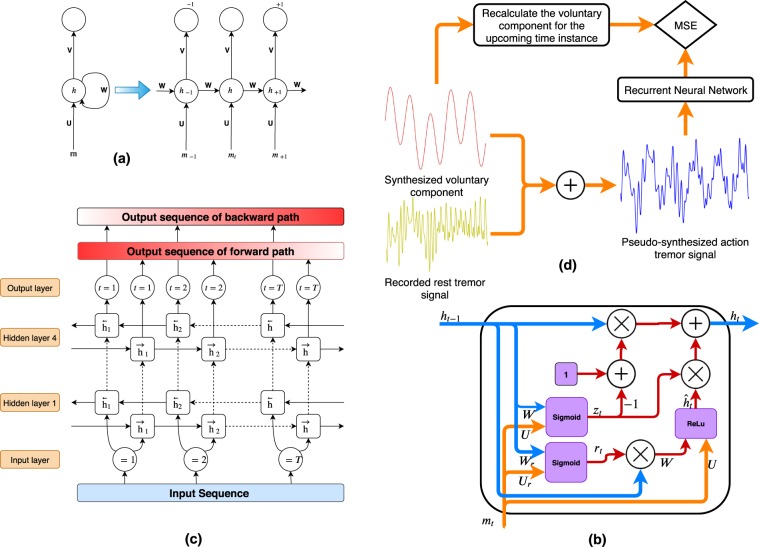
(**a**) A schematic of a one-layer RNN within the PHTNet with the unfolded version demonstrated on the right-hand side, which clearly shows the processing pipeline for different time instances. For the schematic on the left-hand side, it should be noted that the branch denoted by the weight ***W*** also applies one sample delay in time. **(b)** A gated recurrent unit (GRU) which is employed in PHTNet as the recurrent cell and is equipped with reset gate (*r*) and update gate (*z*). **(c)** The architecture of PHTNet, which is a 4-layer deep bidirectional recurrent neural network. $\overleftarrow{h}$ defines the backward cells for offline tremor elimination, and $\overrightarrow{h}$ defines the forward cell of the network for online tremor estimation/prediction. As shown in the diagram, the forward path is completely distinct from the backward path and their outputs are not merged into a single output sequence. The red gradient in the output blocks represents the degree of error in the extracted voluntary component. The high intensity of the red color symbolizes a high degree of error and the opposite mimics lower error rates. **(d)** The overall workflow of the proposed framework. Note that the voluntary component is recalculated for the next time instance and then is compared with the output of the network. This strategy is taken to enable the network with predictive features.

Recurrent Neural Network (RNN) models are a subcategory of representation learning methods^63^ which are specialized in analyzing sequential data and detecting long-term and short-term temporal dependencies in signals based on nonlinear embedded memory. An RNN model consists of a sequence of hidden cells employed to process a stream of data. In RNN models, at each time instance, a combination of input sequence, i.e., hand motion signal, and hidden state vector of the previous time instance are analyzed together to update the state vector and pass it to the next time instance. This process continues until the whole sequence of hand motion measurements is analyzed and a meaningful representation is formed. RNNs have various designs to fit different applications, e.g., sequence to sequence RNNs are employed for machine translation tasks, and sequence to single-output RNNs are employed for classification tasks. Since in this work, we translate a tremor-contaminated sequence to a voluntary component sequence, we employ the sequence-to-sequence architecture. A typical RNN representation is depicted in Fig. 2a and the formulations governing the RNN are given by

2 $${\boldsymbol{h}}(t)={\rm{f}}({\boldsymbol{b}}+{\boldsymbol{Wh}}(t-\mathrm{1)}+{\boldsymbol{Um}}({t}_{1}:t)),$$

3 $${\rm{and}}\,\hat{{\boldsymbol{y}}}(t)={\rm{softmax}}({\boldsymbol{c}}+{\boldsymbol{Vh}}(t)),$$

where ${\boldsymbol{m}}({t}_{1}:t)=[m({t}_{1}),\ldots ,m(t{)]}^{T}$ is the hand motion signal from time (${t}_{1} < t$) to time $t$ as the input sequence of the network; ${\boldsymbol{h}}(t)$ is the hidden feature vector; ***b*** is the bias vector for the input nodes; ***W*** is the weight matrix for hidden-to-hidden connections; ***U*** denotes the input-to-hidden weights of the RNN; ***c*** is the bias vector for the output nodes; ***V*** denotes the weight matrix for hidden-to-output connections; and f($\cdot$) denotes a nonlinear function; here the Rectified Linear Unit (ReLu)^64^ is employed. We note that the weights and biases in Eqs. (2–3) are derived/optimized during the training phase. The schematic of a GRU cell is shown in Fig. 2b.

Using an RNN it can be expected that the output of the network for the very initial input samples is inaccurate and as the information propagates across the network and more samples of the input sequence are analyzed, the output becomes more accurate. Consequently, the output sequence becomes more reliable (in terms of its similarity to the ground truth signal) after a transient phase of initial inputs. As in this work, our goal is to develop an online and offline tremor extraction framework, we structure the processing pipeline in a bidirectional format which employs two parallel sets of recurrent cells for the two processing schemes. As shown in Fig. 2c, forward cells are employed for online (predictive) processing of the input sequence where we need maximum accuracy of estimation for the last samples of the output sequence. Backward cells, on the other hand, are employed for offline processing of measurement signals, following the same logic for the forward cells. It is worth mentioning that in the utilized architecture, the base of which has been named in the literature as Bidirectional RNN (BRNN)^65^, the forward and backward hidden cells are usually followed by a mixing matrix which merges the outputs of the two paths. However, in this work, through an architectural modification of the model, the BRNN kernel is applied without the mixing matrix. This is done since the ultimate goal is to have two separate processing pipelines for both online and offline applications. Finally, Fig. 2d shows the devised training strategy to teach the network how to estimate/predict the voluntary motion.

A common problem with the classical versions of the RNN model described in Eqs. (2 and 3) is its weakness in capturing long-term patterns of the input sequence. This shortcoming is pronounced when long sequences of data need to be processed or when the input sequence encapsulates nonstationary patterns, which is the case for PHT extraction. Moreover, training of these networks is very critical since the problems of vanishing or exploding gradients are prevalent. To address these issues, two gates namely “reset gate” and “update gate” were adopted from the literature and integrated into the conventional hidden cells, and Gated Recurrent Unit (GRU) cells^66^ were developed. The reset gate determines the degree of dismissing old information and considering the data from input in the current time. The update gate, on the other hand, defines the degree of updating a hidden state based on the newly arrived data^67^. Therefore, we can update Eq. (2) as follows

4 $${\boldsymbol{r}}=\sigma ({{\boldsymbol{U}}}_{r}{\boldsymbol{m}}({t}_{1}:t)+{{\boldsymbol{W}}}_{r}{\boldsymbol{h}}(t-\mathrm{1)),}$$

5 $$z=\sigma ({{\boldsymbol{U}}}_{z}{\boldsymbol{m}}({t}_{1}:t)+{{\boldsymbol{W}}}_{z}{\boldsymbol{h}}(t-\mathrm{1)),}$$

6 $$\tilde{{\boldsymbol{h}}}(t)={\rm{ReLU}}({\boldsymbol{Um}}({t}_{1}:t)+{\boldsymbol{W}}({\boldsymbol{r}}\odot {\boldsymbol{h}}(t-\mathrm{1))),}$$

7 $${\rm{and}}\,{\boldsymbol{h}}(t)=\mathrm{(1}-z){\boldsymbol{h}}(t-\mathrm{1)}+z\tilde{{\boldsymbol{h}}}(t),$$

where the reset gate is denoted by *r* and the update gate is denoted by $z$. Consequently, their corresponding weights are denoted by ***U***_*r*_, ***W***_*r*_ and ***U***_*z*_, ***W***_*z*_, respectively. The term $\sigma$ denotes a logistic sigmoid function. As PHT can be a highly dynamic and nonstationary phenomenon, GRU cells are utilized in this work to better capture the long-term behavioural variations of the hand motion signal.

To conclude this part, we have employed a modified deep BRNN to process the hand motion signals and to estimate and predict the voluntary motion of patients. It should be added that deep learning methods, which are a subcategory of representation learning techniques, are composed of several levels of simple but nonlinear units. Each level transforms and abstracts the raw input to a point where complex functions are learned^62^. The proposed PHTNet is a deep architecture constructed by stacking four BRNN layers such that the output of one layer is provided as the input to the next layer.

### Proposed geometry of the PHTNet

**Figure 3:**
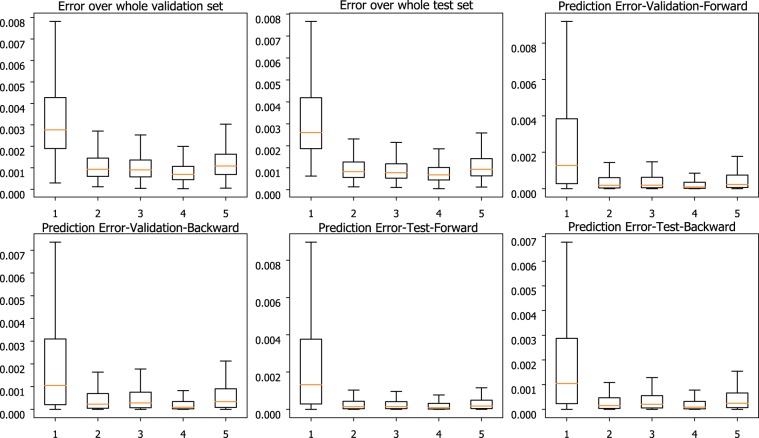
Comparison of normalized MSE (y-axis) over validation and test sets for different number of hidden layers (x-axis). Performance of the PHTNet in each test case is shown in boxplots, where the orange line indicates the median, the box indicates the range between 25% to 75% quartiles, and the lines indicate the standard deviation range. “Prediction Error” indicates the error for only the last sample of estimated voluntary signal. “Forward” and “Backward” indicate which path of the PHTNet is used.

**Figure 4:**
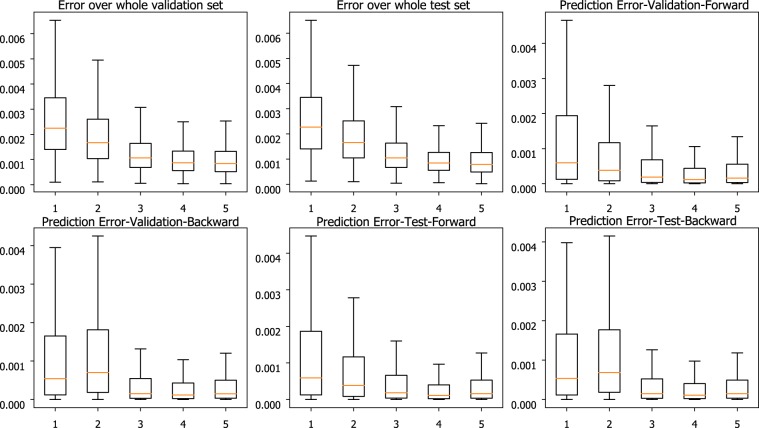
Comparison of Normalized Mean Squared Error (y-axis) over validation and test sets for different lengths of input sequence in seconds (x-axis). Performance of the PHTNet in each test case is shown in boxplots, where the orange line indicates the median, the box indicates the range between 25% to 75% quartiles, and the lines indicate the standard deviation range. “Prediction Error” indicates the error for only the last sample of estimated voluntary signal. “Forward” and “Backward” indicate which path of the PHTNet is used.

Rigorous performance validation of PHTNet is satisfied by grid-searching over potential hyperparameters of the network, i.e., the length of input sequence, the number of RNN features, the number of hidden layers in the deep architecture, and the learning rate for the optimization algorithm. To identify the number of hidden layers in the RNN architecture, a comprehensive grid-search approach is taken to compare the MSE value over validation and test sets across different number of hidden layers. In this regard, the error of network over validation and test sets is obtained and plotted as a function of the number of hidden layers. As shown in Fig. 3, the best performance of the PHTNet is achieved when 4 hidden layers are stacked to each other. In PHTNet, 4 GRU cells are used to process the input sequence. To select the length of the input signal to be fed to the PHTNet with the aim of maximizing the overall performance of the network, we have conducted a comprehensive grid-search approach to evaluate and compare effects of using different lengths of the input signals. To this aim, we have investigated performance of the PHTNet in terms of normalized Mean Squared Error (MSE) over 24,300 validation samples in 5 cases, where the input signal length is set to 1, 2, 3, 4, and 5 seconds for each case. The results of this experiment are shown in Fig. 4. As it can be observed, performance of the PHTNet improves as the length of the input signal increases to a certain point and then either degrades or remains, more or less, unchanged. It is worth mentioning that while performance of the PHTNet remains almost the same as we increase the length of the input signal beyond 4 seconds, computational cost of running the algorithm will increase. The PHTNet, therefore, yields the best performance when input sequences of 4 seconds are fed into the network considering jointly accuracy and computational cost in perspective. Moreover, and based on our rigorous validation procedure, it turned out that using 400 features for ***h***(*t*) in Eq. (7) best abstracts and represents the motion signal in terms of providing maximum estimation accuracy of the tremor component. The network is trained based on minimizing the Mean Squared Error (MSE) value, and the ADAM Optimizer^68^ with a learning rate of 0.0001 employed for this purpose (the learning rate defines the degree of update for the parameters of a neural network in the training session).

As stated previously, providing predictions on the estimated voluntary component of hand motion signals is an important feature for robotic rehabilitation technologies and an unmet need in the literature. A contributing factor for the absence of this feature is the highly dynamic behavior of PHT in and across affected individuals. Predictive operation of a PHT elimination framework grants the robotic systems enough time to adjust their parameters for the subsequent tremulous events. To address this, we have modified the way we feed the training examples to the network. In fact, instead of normally feeding PHTNet with ${\boldsymbol{m}}({t}_{1}:t)$ and calculating the MSE value between $\hat{{\boldsymbol{y}}}({t}_{1}:t)$ and ${{\boldsymbol{m}}}_{v}^{({\rm{GT}})}({t}_{1}:t)$, we measure the estimation error between $\hat{{\boldsymbol{y}}}({t}_{1}:t)$ and ${{\boldsymbol{m}}}_{v}^{({\rm{GT}})}({t}_{1}+\mathrm{1:}t+\mathrm{1)}$ to train PHTNet. In other words, and as depicted in Fig. 2d, our devised strategy is to minimize the error between the network’s output and a shifted version of the ground truth signal, which teaches the network how to estimate the voluntary component of hand motion and also to predict the upcoming future samples of the voluntary component. It is worth emphasizing that to enable the PHTNet with predictive behaviour, no translational parameter or hyper-parameter is considered and this feature is taught to the network by our devised training strategy. In other word, this behaviour becomes an intrinsic characteristic of the PHTNet. For this, when the network is trained to minimize the error between the input sequence and the advanced-in-time output sequence, it is actually learning how to predict the voluntary action in time. To clearly itemize the step-by-step training procedure of the PHTNet, its algorithmic workflow is summarized in Algorithm 3. Please note that the PHTNet could be employed as a plug-and-play model in practical applications and the itemized steps in Algorithm 3 only describe the development phase of the PHTNet.

**Algorithm 1:**
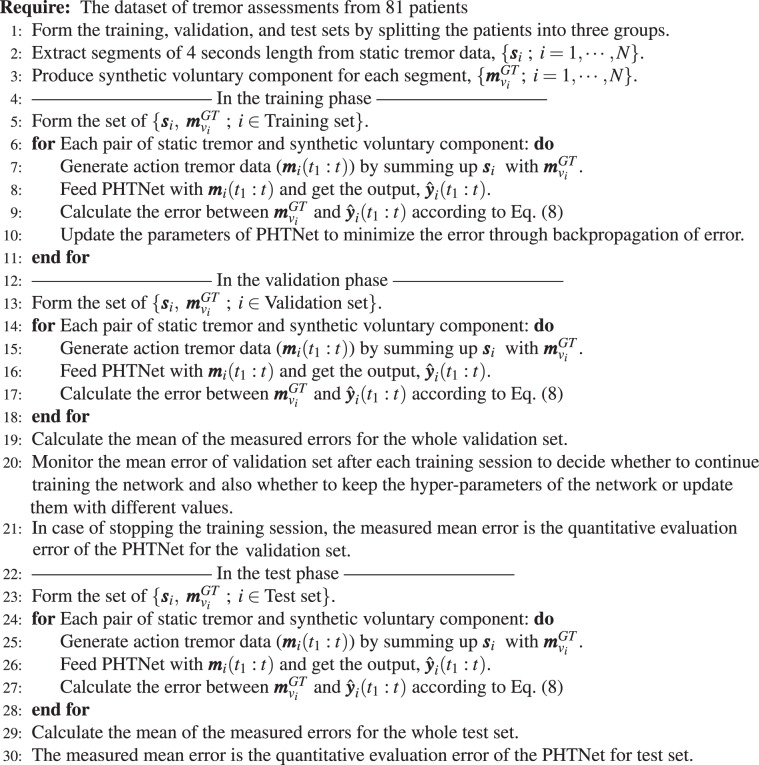
The algorithmic overview of the development phase of the PHTNet.

### Evaluation metrics

In this work, we argue that the existing techniques for PHT elimination may suffer from the absence of a generalizable and inclusive method of extracting the ground truth for the voluntary component of action tremor to reliably measure the performance of the system. Hence, instead of employing conventional methods to extract the ground truth and calculate the performance, an inverse evaluation method is implemented. To this end, pseudo-synthesized action tremor signals are generated by mixing real static tremor recordings with a synthesized atlas of voluntary components, which provides an opportunity to numerically assess the performance measurement of PHTNet in training, validation, and test phases, without specifying assumptions to extract the ground truth signal. Existing methods on PHT elimination show limited performance in estimating the voluntary component of motion signals due to their dependence on different assumptions on the characteristics of the tremor, which normally shows a high degree of inter- and intra-subject variability. As our devised solution for PHT elimination is based on data-driven methods, and we can potentially generate any possible foreseeable signal for voluntary movements, we believe that employing pseudo-synthesized signals adequately addresses this challenge and eliminates the need for making unrealistic assumptions on tremor behaviour. To quantitatively measure the performance of PHTNet, the MSE criterion was used:

8 $${\rm{MSE}}=\frac{1}{T}\mathop{\sum }\limits_{t\mathrm{=1}}^{T}{({m}_{v}^{({\rm{GT}})}(t)-{\hat{y}}_{v}(t))}^{2},$$

where ${m}_{v}^{({\rm{GT}})}(t)$ is the ground truth for the voluntary component, ${\hat{y}}_{v}(t)$ is the estimated voluntary component, and $T$ is the length of the input sequence to the network. We have employed the MSE criteria in various evaluation scenarios to fully investigate the performance of PHTNet.

Moreover, we have employed a qualitative approach, to visually investigate the performance of PHTNet in PHT elimination and demonstrate its superior estimation accuracy compared to its counterparts. In visual inspection, we can verify if the output of PHTNet follows the low frequency trend of the measurement signal, i.e., the voluntary component, that we intuitively expect. The visual investigation is performed to check if the designed neural network is operating in the expected way. It should be noted that visual inspection is not a means of evaluating the performance of PHTNet and only serves as the preliminary verification of the network.

## Results

In this section, the performance of PHTNet is evaluated in several scenarios. Also, supporting results on the effectiveness and capacity of the devised PHTNet for PHT extraction problem are also discussed.

### Quantitative evaluation

**Figure 5:**
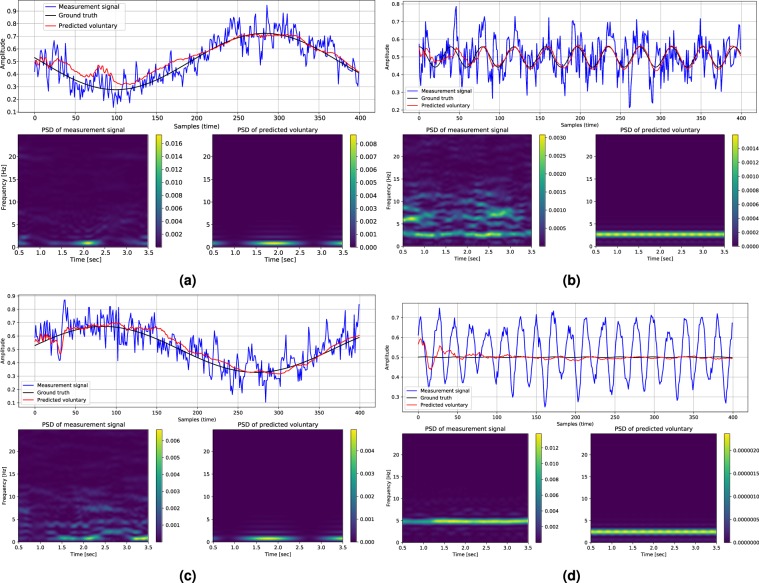
Visualization of the network’s output when the pseudo-synthesized evaluation signals from validation and test sets are fed to the network. Please note that due to the pseudo-synthesized nature of these signals, the known synthetic ground truth is mixed with pathological data to augment the input space and enforce the model to learn how to extract the pathological tremorous motion. The PSD of input and output signals are also included to demonstrate the transformation and manipulation of the spectral contents. The PSD plots are obtained by sliding a Fast Fourier Transform (FFT) window of size 50 samples and overlap size of 45 samples over the signals. The color bars also determine the density of each frequency at each time and its dimension is $\frac{Amplitud{e}^{2}}{Hz}$. **(a,b)** Two instances of the synthesized evaluation signals from the patients in the validation set. **(c,d)** Two instances of the evaluation signals generated from the static tremor recordings of patients in the test set. Please also note that the presented PSD diagrams are only obtained for visualization purposes and their properties do not influence any parameter or hyper-parameter of the PHTNet.

Here, the results of a quantitative evaluation over the pseudo-synthesized validation and test sets are reported. As stated previously, the benefit of employing pseudo-synthesized validation and test sets is the possibility of numerical performance evaluation. Static tremor recordings of 16 subjects for validation and 16 subjects for testing are combined with voluntary components to synthesize data for quantitative evaluation. Please note that the tremor assessments of one patient only belong to the training set, or the validation set, or the test set to avoid leakage of data from training set to the other two sets. The network’s output for different pseudo-synthesized inputs is shown in Fig. 5. Please note that the PSD diagrams in Fig. 5 are derived by sliding a Fast Fourier Transform (FFT) window of size 50 samples and overlap size of 45 samples over the signals. A high overlap size is selected to produce a smooth representation of the spectral contents of the signals. It is worth mentioning that the aforementioned properties are only employed for visualization purposes and do not reflect any parameter or hyper-parameter for the PHTNet. It is worth mentioning that to further investigate the performance of PHTNet and to discover the way it manipulates the spectral contents of the input signal, the Power Spectral Density (PSD) of the input and output pairs are also shown. The numerical results of the quantitative tests are presented in Table 2. As the main goal of this work is to develop a PHT estimation framework for online and offline applications, we believe that it is necessary to monitor the estimation error for the forward and backward cells, separately. In addition, as the last samples of the output sequence in the forward direction are mainly employed for online applications, and the initial samples of the output in the backward direction are employed in offline applications, we have also measured the estimation error for the last sample in the forward cells and the estimation error associated with the first sample in the backward cells.

**Table 2:** Results of a quantitative evaluation of the network in different testing scenarios.

	Validation set	Test set
MSE of estimation for a complete segment	$0.00111$	$0.00104$
MSE of estimation for the last sample of segment over 24300 trials (*forward cells*)	$0.00056$	$0.00049$
MSE of estimation for the first sample of segment over 24300 trials (*backward cells*)	$0.00052$	$0.00048$

**Figure 6:**
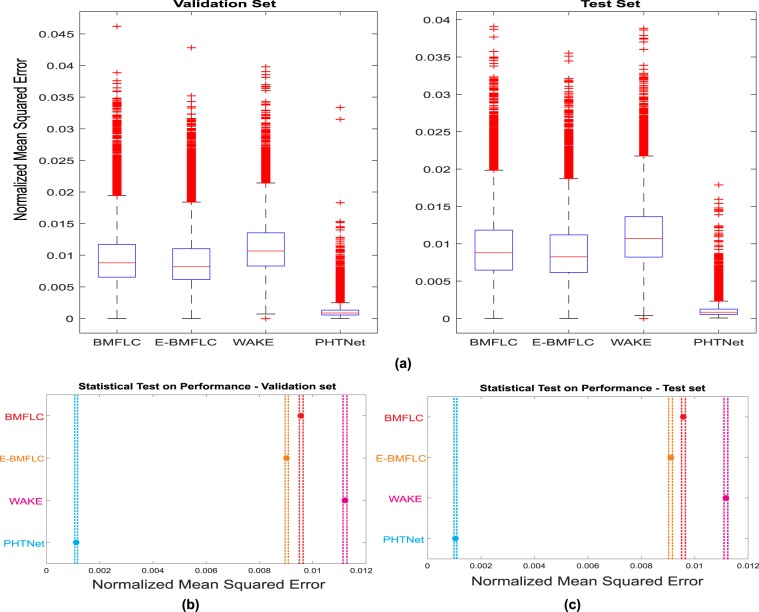
(**a**) Comparison of the performance between BMFLC, EBMFLC, WAKE, and PHTNet on the validation and test sets. The red line in the boxplots indicates the median performance and the box indicates 25%% and 75% quartiles. The dashed lines show standard deviation and the red crosses show outliers. **(b,c)** Multiple comparison of the mean performance of the four methods over the validation and test sets. The dashed lines indicate the upper and lower 95% confidence bounds for the estimation error of each method and the y-axis represents the labels of compared PHT removal techniques.

Although the examples presented in Fig. 5 demonstrate how the spectral contents of the involuntary component are manipulated, a more rigorous analysis is required to take the whole validation and test sets into account and statistically investigate the effectiveness of the PHTNet framework for PHT elimination. To statistically investigate performance of the PHTNet in comparison to the state-of-the-art methods on PHT removal, and due to the fact that we need to have pseudo-synthesized action tremor data to accurately measure the error in estimation, PHTNet along with three well-regarded techniques on PHT removal, i.e., BMFLC, EBMFLC, and WAKE, were examined over the validation and test sets of pseudo-synthesized action tremor. To statistically compare the four methods, the “Analysis of Variance (ANOVA)” test, and pairwise “Z-test” between each two groups are employed, where the results are shown in Fig. 6 and Table 3. As it is understood, PHTNet shows significantly better performance compared to state-of-the-art methods in the literature. It is worth noting that in the boxplots in Fig. 6a, the red line indicates the mean value, the box indicates the range between 25% to 75% quartiles, the black lines indicate the standard deviation range, and red crosses indicate the outliers. Furthermore, Fig. 6b,c show multiple comparison of the mean performance of the four methods over the validation and test sets. The dashed lines indicate the upper and lower 95% confidence bounds for the error of each method. The disjoint confidence intervals of PHTNet with other methods indicates that the mean performance of the PHTNet is significantly different from other methods. It is worth clarifying that the plots in Fig. 6b,c show the mean Normalized MSE (shown as a circle) and the 95% confidence intervals (shown as dashed lines) for each of the four techniques that are evaluated over the validation and test sets. The *y*-axis of the plots represents the label of each technique and the disjoint area between the dashed lines of each two methods verifies that each two methods have significantly different mean performance from each other.

**Table 3:** Comparison of the performance between PHTNet and three well-regarded PHT processing frameworks.

Dataset		BMFLC-PHTNet	EBMFLC-PHTNet	WAKE-PHTNet
Validation	p-value	$5.96E-08$	$5.96E-08$	$5.96E-08$
	Improvement	$+\mathrm{88.42 \% }$	$+\mathrm{87.78 \% }$	$+\mathrm{90.18 \% }$
Test	p-value	$5.96E-08$	$5.96E-08$	$5.96E-08$
	Improvement	$+\mathrm{89.58 \% }$	$+\mathrm{89.01 \% }$	$+\mathrm{91.07 \% }$

The $p-values$ are derived based on 95% confidence intervals. The numbers in the “Improvement” rows represent the improvement in the mean Normalized MSE obtained when moving from other techniques to the PHTNet.

**Figure 7:**
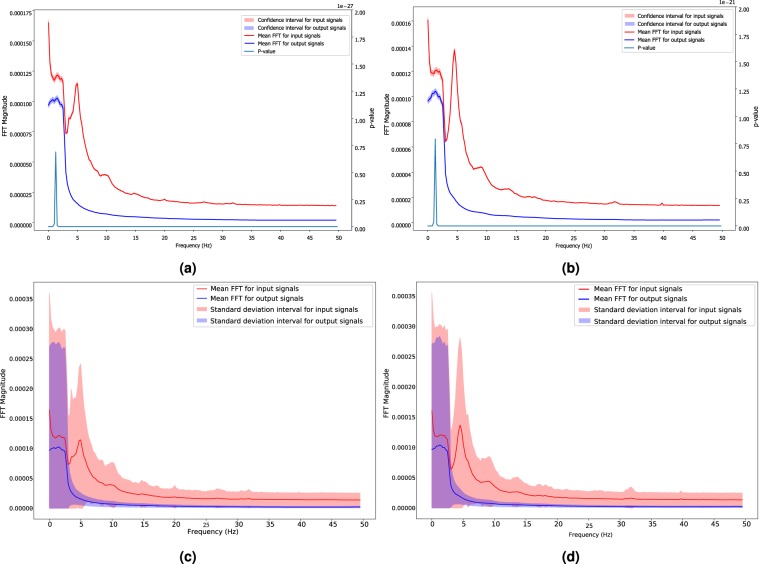
(**a**,**b)** Visualization of the 95% confidence boundaries for the spectral contents of input-output pairs in validation and test sets, respectively. The solid lines indicate the mean of spectral contents and the highlighted area indicate confidence boundaries. Also note that the y-axis on the right represents the $p-value$ between the spectral contents of the input and output signals. **(c,d)** Visualization of standard deviation boundaries for input-output pairs along with the mean of spectral contents of input and output signals in validation and test sets, respectively. Please note that the spectral contents of the input signals are shown in “red” color, while those of the output signals are depicted with “blue” color. The dominant activity at around 5 Hz is only observed in the input signals (“red” color), which is expected due to the strong power of pathological tremor around 5 Hz.

Furthermore, to statistically investigate the efficacy of PHTNet in manipulating the spectral contents of hand motion signals, the FFT of the input and output signals in the validation and test sets are derived and statistically compared. Due to the large number of samples in each of the validation and test sets (24,300 signal pairs for each set), we employed “Z-test” for statistical comparison of the input and output groups. We performed D’Agostino and Pearson’s test^69^ to check if the data samples for each frequency and for each of the input and output groups follow a normal distribution. After verifying the normality of data samples, we employed the “Z-test” to extract the confidence intervals by setting $Z=1.96$, which corresponds to 95% confidence bounds. In fact, the confidence interval states that the probability that the mean of data samples occurs in the range of (−1.69 * *σ*/$\sqrt{n},\,1.96\ast \sigma /\sqrt{n}$) is equal to 0.95, where $\sigma$ and *n* represent the standard deviation and the sample size, respectively. As shown in Fig. 7a,b, the mean and confidence intervals for the input and output pairs in the validation and test sets are completely distinct and the extracted $p-value$ between the input and output pairs, which is $\ll 0.01$ at each frequency, verifies that applying the proposed PHTNet was resulted in a significant change in the spectral information of the signal by removing the tremor, such as the illustrative examples shown in Fig. 5. In addition, Fig. 7c,d show the mean FFT along with its standard deviation intervals (mean $\pm$ standard deviation) for the input-output pairs. Please note that all of the presented figures do not include the spectral content corresponding to 0 Hz to better scale the figures and present a more detailed picture. It is worth noting that the results shown in Fig. 7c,d represent the variability of the spectral contents for each group around the mean spectral value of the population. Although the efficacy of the PHTNet in damping the spectral contents of the PHT is reflected in Fig. 7c,d, it is not readily inferred whether the spectral contents of the input and output populations are significantly different or not. Thus, the plots in Fig. 7a,b are generated to reveal the *p*-value between the two populations at each frequency point and also to illustrate the position of the two spectral populations with respect to each other. As demonstrated in Fig. 7, a significant decrease of power in the spectral content above 3 Hz is observed, which reveals the filtering behaviour of PHTNet, although we did not explicitly design a spectral filter. More importantly, unlike conventional spectral filtering methods which normally result in phase-lag (delay) in their output signals, the proposed PHTNet advances the input signal by predicting the voluntary component in the next time.

### Predictive behaviour analysis

**Figure 8:**
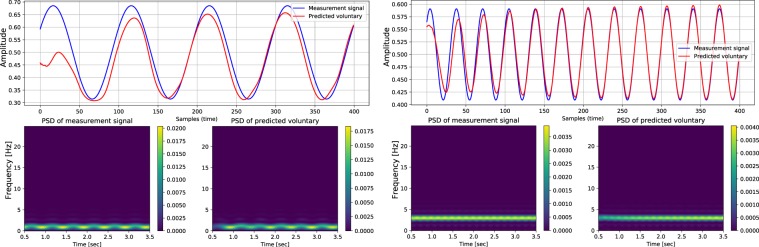
Visualization of the predictive capability of the network over special test signals.

As mentioned earlier, one goal of this work is to predict the voluntary motion of an individual in a one-step-ahead-of-time fashion, which is of significant importance in robotic rehabilitation technologies. To address this goal, we devised a novel training strategy to equip PHTNet with predictive functionalities. Table 2 shows the numerical evaluation of the predictive behaviour of PHTNet over 48600 input-output pairs for both validation and test sets. However, to visually inspect the output signal and verify if it actually advances the input signal, in this part, we employ pure sinusoidal signals as inputs to PHTNet. The benefit of feeding the network with sinusoidal signals is that we can clearly observe the status of the output signal with respect to the input, without any interference from the involuntary component of the hand motion signals. Figure 8 shows the capability of the trained network in predicting the voluntary component.

### Qualitative performance monitoring

**Figure 9:**
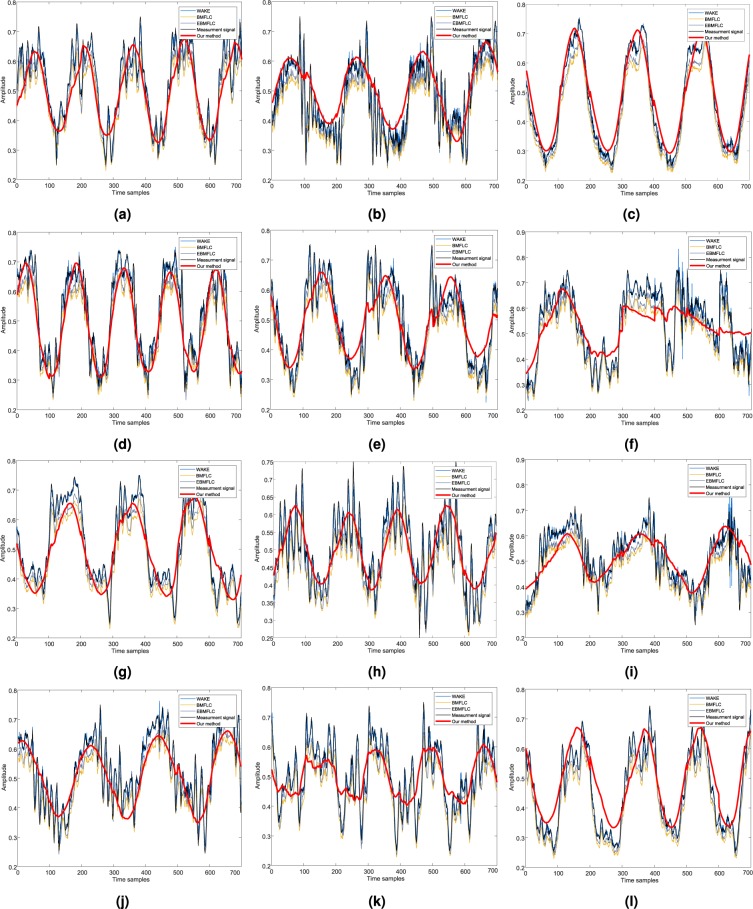
Visualization of the network output when real action tremor signals are fed to the network. Our method was compared with three other methods, referred to as BMFLC, EBMFLC, and WAKE. The details of each patient whose signal is shown here are as follows. **(a)** [ET - Right hand - de novo - Training]. **(b)** [PD - Right hand - Under treatment - Validation]. **(c)** [PD - Left hand - de novo - Test]. **(d)** [ET - Right hand - Under treatment - Test]. **(e)** [PD - Right hand - Under treatment - Test]. **(f)** [PD - Right hand - de novo - Test]. **(g)** [PD - Right hand - Under treatment - Test]. **(h)** [ET - Right hand - de novo - Training]. **(i)** [PD - Right hand - Under treatment - Training]. **(j)** [PD - Right hand - de novo - Training]. **(k)** [ET - Left hand - Under treatment - Validation]. **(l)** [PD - Left hand - Under treatment - Validation].

**Figure 10:**
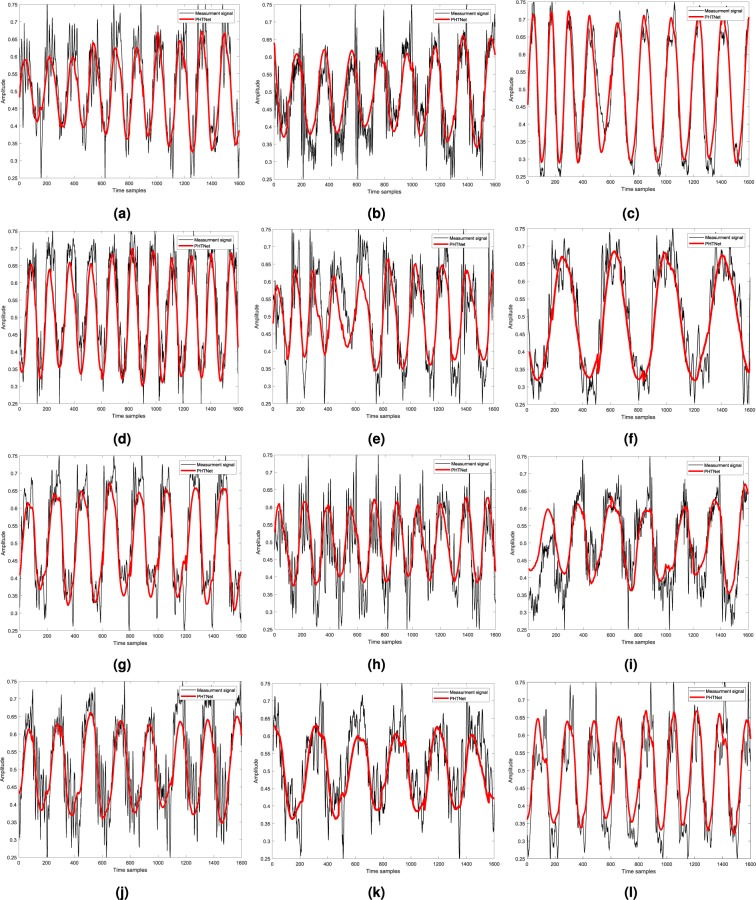
Visualization of the network output when real action tremor signals are fed to the network. In this case, the prediction samples are employed. The details of each patient whose signal is shown here are as follows. **(a)** [ET - Right hand - de novo - Training]. **(b)** [PD - Right hand - Under treatment - Validation]. **(c)** [PD - Left hand - de novo - Test]. **(d)** [ET - Right hand - Under treatment - Test]. **(e)** [PD - Right hand - Under treatment - Test]. **(f)** [PD - Right hand - de novo - Test]. **(g)** [PD - Right hand - Under treatment - Test]. **(h)** [ET - Right hand - de novo - Training]. **(i)** [PD - Right hand - Under treatment - Training]. **(j)** [PD - Right hand - de novo - Training]. **(k)** [ET - Left hand - Under treatment - Validation]. **(l)** [PD - Left hand - Under treatment - Validation].

Qualitative inspection of the PHTNet framework was performed on the action tremor recordings due to the absence of a valid framework to extract the voluntary component of hand motion signals. As the performance of PHTNet over real action tremor signals cannot be numerically reported, performance monitoring is conducted through visual inspection, where one checks if the estimated voluntary component is aligned with the expected low-frequency trend in the signal or not. Figure 9 shows 12 instances of the processed signals which include action tremor recordings from the training, validation, and test sets. To further investigate the performance of the PHTNet framework, we compared our results with BMFLC^51^, EBMFLC^13^, and WAKE^14^. While the FLC-based methods, e.g., BMFLC and EBMFLC, are focused on modeling the spectral contents of the measurement signal with a linear combination of spectral components, the WAKE method employs spectrotemporal techniques and Kalman filtering to decompose the measurement signal into the two components of motion. Moreover, to visually inspect the performance of the forward (predictive) path of the PHTNet over action tremor data, the action tremor signals shown in Fig. 9 are also processed by the forward path and the results are shown in Fig. 10. As it is shown, the output of PHTNet perfectly follows the voluntary component of motion that we visually expect.

### PHTNet over healthy controls

**Figure 11:**
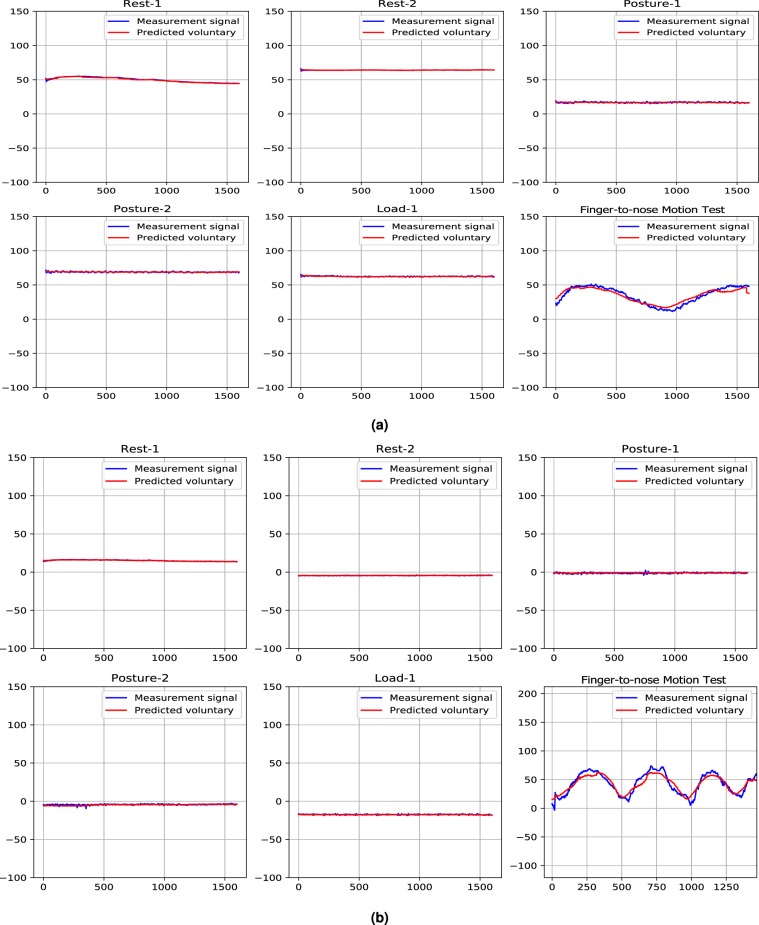
(**a,b**) Results of applying PHTNet on the recordings of static posture tasks and finger-to-nose motion test for the two healthy subjects.

To investigate the performance of the PHTNet over hand motion recordings from healthy individuals, 2 set of signals from 2 healthy volunteers were recorded. Two healthy male individuals aged 28 and 37 participated in the data collection procedure and the acceleration of their hand motion is recorded with Trigno Avanti Wireless System PM-W05 (Delsys Inc.). Trigno Avanti sensors have a built-in 9 DOF inertial measurement unit which can relay acceleration, rotation and earth magnetic field information. The sensitivity of the sensor is set to $\mp 16\,g$ and the sampling rate by default is 370.37 Hz. However, for this experiment the recordings are downsampled to 100 Hz to match the training data of the PHTNet and the results are shown in Fig. 11. Performing this test is crucially important to check the performance of the PHTNet over hand motion recordings without tremor component. As it is observed in the figure and was expected before implementing the test, the estimated signals by PHTNet perfectly match the input signals, as no manipulation should be applied on signals without tremor component. It should be noted that the kinematic data for the hand from healthy individuals was recorded with a different apparatus from that collected in the original dataset. While this was due to an unforeseen situation whereby the original equipment was not available, we believe that the use of a different but clinically accepted device to assess the functionality of the PHTNet provides a good opportunity to examine the generalization of the network and its level of independence of the recording device. In fact, the results shown in Fig. 11 not only show the flawless performance of the PHTNet in processing the kinematics of hand but also reflect a high degree of generalization over the characteristics of PHT and the independence of the PHTNet with regard to the recording device.

## Discussion

From the results of the experiments over the validation and test sets presented in Figs. 5 and 9, and Table 2, we can clearly observe the superior performance of PHTNet in accurate estimation of the voluntary hand motion from pseudo-synthesized and real action tremor signals. The examples presented in Fig. 5 include different possible cases for voluntary component, i.e., [high vibration amplitude - low frequency] in Fig. 5a,c; [low vibration amplitude - high frequency] in Fig. 5b, and [very low (near zero) vibration amplitude] in Fig. 5d. Observing the PSDs of input-output pairs also clearly shows how the high frequency components are damped, while at the same time, the low frequency trend in the measurement signal is magnified. RNNs typically yield inaccurate outputs for the first few samples of the input sequence and as more information is fed into the network, the estimation process becomes more accurate. This natural behavior of RNNs is also observed in the validation examples shown in Fig. 5, which confirms the necessity to employ a bidirectional architecture with two separate processing pipelines within the PHTNet. Thus, the bidirectional architecture empowered the PHTNet to maintain the required level of accuracy for PHT elimination tasks, when it is used for online applications through forward cells, and for offline applications via its backward cells. It is important to note that the instances presented in Fig. 5 are derived by employing the forward cells of PHTNet. To evaluate the network in online and offline applications, the estimation accuracy is shown in Table 2 for the next time sample when forward cells and backward cells are employed. The results clearly show the accuracy of PHTNet in estimating the voluntary component, when enough information is fed to the network. In addition to the instances shown, a rigorous statistical analysis is performed on all of the samples in the validation and test sets to fully examine the operation of PHTNet. In this regard, standard deviation and 95% confidence boundaries were calculated over 24, 300 samples in the validation and test sets, as shown in Fig. 7. These results suggest that PHTNet operates as a low-pass spectral filter; however, the predictive behaviour of PHTNet makes it distinct from any previously known spectral filter. In Fig. 7, a dominant activity at around 5 Hz is observed in the spectral domain of input signals (“red” color), which verifies the reported characteristics of the PHT in the literature, i.e., the PHT in PD and ET patients commonly occurs in 4–6 Hz and 4–8 Hz^17,70^, respectively, which results in accumulation of power in frequencies around 5 Hz. In the qualitative performance monitoring part, the output of PHTNet is compared with well-regarded, recent works in the field of PHT estimation, i.e., BMFLC, EBMFLC, and WAKE. As shown in Fig. 9, our proposed method provides a smooth and tremor-free output, which is compatible with the visual trend that we expected, and is also robust to sudden high amplitude vibrations. Please note that for the instances presented in Fig. 9, the backward cells of PHTNet are employed to extract the voluntary component of hand motion signals. In this setup, the PHTNet slides over the action tremor signal and the estimated voluntary component is obtained. To only keep the accurate part of the output signal, which in this case consists of the first few samples of the estimated signal, PHTNet advances for 50 samples and again the estimation is performed. This process continues until the whole sequence of action tremors is processed. In this context, the network slides over the measurement signal and outputs the voluntary component. To show the generalization of the network, we have included action tremor instances from training, validation, and test sets. To further assess the predictive behavior of the network in addition to the numerical results reported in Table 2, which clearly illustrate the accuracy of estimation when forward cells are employed, we fed the network with pure sinusoidal signals to investigate its response to the inputs and verify if the network shows any predictive behaviour.

This paper proposes the design and implementation of a novel voluntary motion prediction and tremor removal technique that can be used for enhancing assistive devices and clinical settings. Although the proposed trained technique significantly performed better than all existing approaches, it requires relatively stronger computational support to be implemented due to the deep neural structure. It should be noted that the training of the model is completed in this paper, and the model can be used as a ready, plug-and-play trained algorithm without the need for retraining. However, utilization of any deep neural network with memory gates, requires sufficient computational power, which can be a limiting factor if the computational resources are strictly limited. Thanks to the power of new processing technologies, this challenge will not be very concerning but should be considered when implementing. In addition, we are working on a cloud computing approach for this work, which can be used for minimizing the need for having on-site computational power. Also, in order to enhance this aspect of the technique, we have an ongoing research to optimize the design of the PHTNet and implement hybrid and shallower models with comparable performance. In addition to the above points, it should be also highlighted that the accurate predictability of the proposed technique is a novel and unique feature, which do not exist in conventional techniques. However, the achieved horizon of prediction was limited. We are planning to augment the input space with other biological modalities and biomechanical models while improving the prediction ability of the technique to further enhance the horizon. Lastly, another future direction for this work would be to expand the size of the dataset employed and investigate the performance of the framework when the kinematics of motion in all dimensions are jointly fed to the network to potentially enhance the perception of the network over the voluntary action and increase its predictive horizon.

## Supplementary information

Supplementary information

## Data Availability

The datasets generated and/or analyzed during the current study are not publicly available due to the confidentiality restrictions imposed by the approved ethics of study but are available from the corresponding author on reasonable request.
