# Supplementary material for: PHTNet: Characterization and Deep Mining of Involuntary Pathological Hand Tremor using Recurrent Neural Network Models
Source: Sci Rep. 2020 Feb 10;10:2195. doi: 10.1038/s41598-020-58912-9 (PMC7010677; doi:10.1038/s41598-020-58912-9)
Supplement: Supplementary file 1 — Supplementary information [file 41598_2020_58912_MOESM1_ESM.pdf]

Title: PHTNet: Characterization and Deep Mining of Involuntary Pathological Hand Tremor using Recurrent Neural Network Models (SREP-19-31658)

Authors: Soroosh Shahtalebi, S. Farokh Atshzar, Olivia Samotus, Rajni V. Patel, Mandar S. Jog, Arash Mohammadi

Document: Demographics of Dataset

Participant ID	Gender	Age (years)	Number of arms assessed	Number of visits	Levodopa daily dose (mg)	FTM part A* (/12) Right hand	Left hand
1	M	71	1	2	N/A	6	0
2	F	65	1	2	400	0	0
3	M	74	1	2	N/A	2	0
4	M	67	2	2	N/A	6	4
5	F	55	1	2	N/A	0	5
6	M	61	2	2	1000	7	6
7	F	78	1	2	N/A	0	3
8	F	81	2	2	N/A	3	3
9	M	80	1	2	500	2	6
10	M	74	2	2	400	6	4
11	M	77	1	2	800	0	4
12	M	73	1	2	400	2	6
13	M	70	1	2	600	2	2
14	M	70	1	2	N/A	3	3
15	M	81	2	2	400	4	3
16	F	73	1	2	N/A	7	1
17	M	67	2	2	435	7	6
18	M	83	1	2	800	5	1
19	M	64	1	2	N/A	2	8
20	M	57	1	2	N/A	2	1
21	M	78	1	2	N/A	3	0
22	M	75	1	2	750	6	0
23	M	60	1	2	400	0	3
24	M	63	2	2	750	4	2
25	M	66	1	2	N/A	1	3
26	M	85	2	2	600	2	4
27	M	67	1	2	400	0	4
28	M	80	2	2	850	4	5
29	F	63	1	2	N/A	5	0
30	M	87	2	2	N/A	5	2
31	F	60	1	2	N/A	4	0
32	M	79	2	1 #	N/A	1	2
33	M	79	2	1 #	N/A	6	4
34	M	69	2	2	300	4	4
35	M		1	2	400	1	4
36	M	64	1	2	N/A	0	5
37	M	67	1	1	N/A	8	2
38	F	77	2	2	N/A	4	2
39	M	83	1	2	200	3	2
40	M		1	2	N/A	5	2
41	M	71	1	2	N/A	4	3
42	M	71	1	2	N/A	4	0
43	M	67	1	2	N/A	2	0
44	M	72	1	2	400	2	1
45	M	69	1	2	N/A	6	1
46	M	75	1	2	N/A	1	6
47	M	70	1	2	800	0	5
Mean	8 F		14 bilateral		551.7; 26 de-novo patients	3.2	2.8
Standard Deviation					217.4	2.3	2.1
Range (low)					200	0	0
Range (high)					1000	8	8

# only has motor-dominant hand tremor analysis for visit 2

* assessment of tremor during rest, posture and action tremor

							FTM part A* (/12)	
Participant ID	Gender	Age (years)	Number of arms assessed	Number of visits	Motor-dominant Hand	Tremor medication list (total daily dose)	Right hand	Left hand
1	M	76	2	2	R	N/A	1	2
2	F	73	2	2	R	N/A	1	4
3	M	63	2	2	R	N/A	6	9
4	M	64	2	2	R	Primidone (750mg)	6	4
5	F	76	2	2	R	(500mg), Propranolc	5	6
6	M	72	2	2	L	N/A	4	3
7	F	69	2	2	R	(500mg), Propranolc	6	9
8	M	70	2	2	R	N/A	6	6
9	M	68	2	1	R	N/A	1	1
10	F	65	2	2	R	N/A	5	2
11	F	79	2	2	R	Propranolol (120mg)	2	2
12	M	67	2	2	R	Propranolol (500mg)	6	8
13	F	66	2	1	L	Propranolol (250mg)	5	3
14	M	68	2	2	R	N/A	4	4
15	F	52	2	2	R	N/A	3	5
16	F	61	2	2	R	Gabapentin (900mg)	3	3
17	F	77	2	2	R	N/A	3	3
18	F	75	2	2	R	N/A	4	2
19	M	75	2	2	R	N/A	4	3
20	F	63	2	2	R	Gabapentin (900mg)	4	3
21	M	78	2	2	R	N/A	4	3
22	M	69	2	2	R	N/A	2	3
23	M	71	2	2	R	Primidone (250mg)	5	6
24	M	71	2	1	R	N/A	1	1
25	M	77	2	2	R	N/A	4	5
26	F	65	2	2	R	Primidone (500mg)	4	5
27	M	74	2	2	R	Propranolol (80mg)	4	3
28	M	83	2	2	R	N/A	6	0
29	M	70	2	2	R	N/A	8	3
30	M	71	2	2	R	N/A	4	7
31	M	70	2	2	L	N/A	5	4
32	M	65	2	2	R	Topiramate (100mg)	4	5
33	F	65	2	2	R	N/A	4	3
34	M	64	2	2	R	N/A	5	6
Mean	13 F	69.8	All bilateral		3 L	22 de-novo patients	4.1	4.0
Standard Deviation		6.2					1.7	2.2
Range (low)		52					1	0
Range (high)		83					8	9

* assessment of tremor during rest, posture and action tremor
